# Exercise delays aging: evidence from telomeres and telomerase —a systematic review and meta-analysis of randomized controlled trials

**DOI:** 10.3389/fphys.2025.1627292

**Published:** 2025-06-26

**Authors:** Liang Sun, Tingran Zhang, Lanfang Luo, Yi Yang, Chuanqiushui Wang, Jiong Luo

**Affiliations:** ^1^ School of Physical Education, Southwest University, Chongqing, China; ^2^ School of Physical Education, Chongqing Mining Engineering School, Chongqing, China

**Keywords:** exercise, aging, telomeres, telomerase, meta analysis

## Abstract

**Objective:**

To systematically evaluate the regulatory effects of exercise intervention on telomere length (TL) and telomerase activity (TA), and to provide evidence for formulating precise exercise prescriptions based on telomere protection.

**Methods:**

Databases including China National Knowledge Infrastructure, Wanfang, VIP, PubMed, Web of Science, Cochrane Library, and Embase were searched to collect randomized controlled trials (RCTs) regarding the regulation of TL and TA by exercise intervention up to February 2025. The Cochrane risk assessment tool was used to evaluate the quality of the included literature. Meta-analysis, heterogeneity test, subgroup analysis, sensitivity analysis, univariate meta-regression analysis, and publication bias test were conducted using Review Manager 5.3 and Stata 18.0 software.

**Results:**

Exercise intervention significantly maintained TL (SMD = 0.59, 95% CI: 0.14–1.06, P = 0.01) and enhanced TA (SMD = 0.35, 95% CI: 0.20–0.51, P < 0.00001). A single study suggests high-intensity interval training (HIIT) may maintain TL (SMD = 0.66, P = 0.01), but this requires further validation due to limited evidence. Aerobic exercise (AE) consistently increased TA (SMD = 0.33, P = 0.0001), while resistance exercise (RE) showed non-significant trends (SMD = 0.16, P = 0.43). Subgroup analysis by sex showed a trend toward greater TL maintenance in females (SMD = 0.48, P = 0.06) compared to males (SMD = 0.38, P = 0.40). An exercise duration of ≥16 weeks was necessary for significant effects. High heterogeneity (I2 = 92% for TL) was partially explained by measurement methods, age, and baseline health.

**Conclusion:**

Exercise maintains TL and enhances TA, potentially contributing to delayed aging. AE shows robust effects on TA, while HIIT and RE require further research due to limited studies. Future studies should standardize measurement methods and explore confounders like diet and genetics.

**Systematic Review Registration:**

PROSPERO, identifier CRD420251006569.

## 1 Introduction

Research indicates that the proportion of the world’s population aged 60 and above is increasing rapidly. It is projected that by 2050, this proportion will rise by 20%, surpassing the number of children globally. This phenomenon suggests that the population structure of most countries is tending towards aging (Stambler, 2017). Therefore, developing interventions that can slow down the aging process or reduce the incidence of aging-related diseases has become an urgent task, which also holds significant application value in improving the quality of life and reducing medical costs (Chakrabarti and Mohanakumar, 2016; Konar et al., 2016). Studies on human and animal models have shown that various genetic, dietary, exercise, and drug interventions can extend lifespan. Meanwhile, these lifespan - extending methods also contribute to delaying the onset of age - related diseases (Kenyon, 2010; Tacutu et al., 2013). In recent years, research has revealed the importance of telomere length (TL) and its integrity in the aging process, as well as potential interventions to delay aging, such as physical exercise and a healthy diet (Mercken et al., 2012). Since TL plays a crucial role in cellular aging and telomere shortening is associated with a decrease in life expectancy and an increased risk of chronic diseases, telomere attrition has been described as one of the important biological features of aging (López-Otín et al., 2013).

Telomeres are special structures at the ends of linear chromosomes, composed of repetitive G - and C - rich DNA sequences (5’ - TTAGGG - 3’/3’ - CCCTAA - 5′) and bound to a protein complex (shelterin), including telomeric repeat binding factor 1 (TRF1), telomeric repeat binding factor 2 (TRF2), protection of telomeres 1 protein (POT1), TRF1 - and TRF2 - interacting nuclear protein 2 (TIN2), TIN2 and POT1 interacting protein 1 (TPP1), and repressor activator protein 1 (RAP1). These proteins directly recognize telomere sequences and assist in forming T - loop and D - loop structures, thus hiding the telomere ends and suppressing the DNA damage response, preventing the activation of ataxia - telangiectasia mutation (ATM) and RAD3 - related (ATR) kinases (Balan et al., 2018; Blackburn et al., 2015; de Lange, 2005). Telomeres play a key role in stabilizing chromosomes, preventing DNA degradation and end - to - end fusion, and regulating cell growth. Simultaneously, as a mitotic clock, their length gradually shortens with cell division, serving as an indicator of cellular replication potential (Arnoult and Karlseder, 2015; Blackburn, 2010). With aging, telomere shortening leads to functional impairment, triggering genomic instability, cell senescence, and apoptosis (Blackburn et al., 2015). Biological aging is a process independent of chronological aging, which reduces the organism’s viability and increases vulnerability. TL, as a biomarker of biological aging, records both chronological and biological age (Brown et al., 2017). When TL shortens below a threshold, it can trigger chromosome fusion, genomic instability, and DNA damage, resulting in the production of non - functional proteins (Cleal et al., 2018; Hemann et al., 2001). These proteins may induce apoptosis or promote cancer development. Although telomere shortening can suppress tumors, its functional loss accelerates cell aging and tissue degeneration, driving organismal aging (Vakonaki et al., 2018). Therefore, maintaining TL is crucial for delaying aging.

Telomerase is an RNA - dependent DNA polymerase composed of telomerase reverse transcriptase (TERT) and telomerase RNA template (TERC), which can provide cells with unlimited proliferation potential by lengthening telomeric DNA (Blackburn, 2001; Cong et al., 2002). Due to the “end - replication problem”, the telomeres of somatic cells gradually shorten with age, while telomerase can slow down this process (Harley et al., 1990; Beyne-Rauzy et al., 2005). The polymorphism of TERT is associated with a reduced risk of breast cancer (Helbig et al., 2017), and telomerase plays a key role in maintaining genomic stability by synthesizing telomeres and counteracting telomere erosion (Zhang F. et al., 2016). In addition, the regulation of telomerase activity (TA) has potential value in anti - aging and cancer treatment (Cong et al., 2002; Aviv, 2002).

With the change of lifestyle, the lifespan and quality of life of the elderly have improved, especially with regular physical exercise. However, the underlying mechanisms remain unclear, which has, to some extent, promoted research on the relationship between exercise and telomere biology, such as whether exercise can delay aging and improve diseases. This systematic review and meta - analysis aim to integrate existing clinical studies and systematically evaluate the regulatory effects of exercise intervention on TL and TA, providing evidence - based support for formulating precise exercise prescriptions based on telomere protection.

## 2 Methods

This study was preregistered at PROSPERO (CRD420251006569) and adheres to PRISMA guidelines.

### 2.1 Literature inclusion and exclusion criteria

Inclusion criteria: Randomized controlled trials (RCTs) from database inception to February 2025, with no baseline differences between experimental and control groups. The control group maintained a regular lifestyle without exercise, while the experimental group received exercise intervention (minimum 16 weeks, ≥60 min/week). Outcome indicators: TL and TA.

Exclusion criteria: Non-RCTs, studies with ineligible outcomes (e.g., animal studies), exercise combined with diet or other interventions, no control group, non-continuous exercise, duplicated publications, or exercise perception training.

### 2.2 Literature search strategy

**FIGURE 1:**
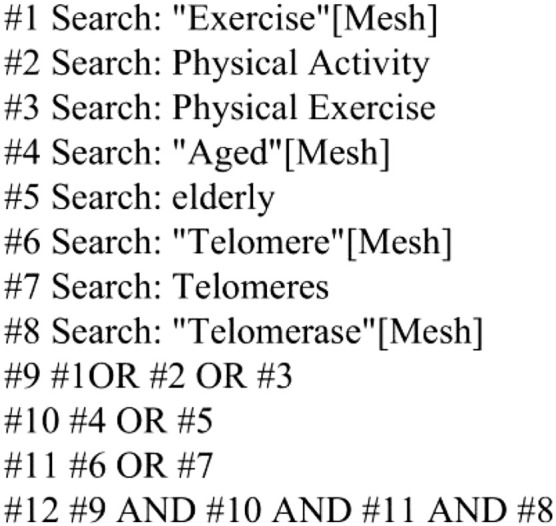
PubMed database search strategy.

Databases (PubMed, Web of Science, Cochrane Library, Embase, CNKI, Wanfang, VIP) were searched using terms “telomeres, telomerase, exercise, senescence” up to February 2025. The PubMed search strategy is shown in Figure 1.

### 2.3 Data extraction

Data were extracted on author, publication year, participant characteristics, sample size, intervention details (time, frequency, method), cell/tissue types, measurement methods, and outcomes. Ineligible studies were excluded after title/abstract or full-text review.

### 2.4 Quality evaluation

The Cochrane risk assessment tool evaluated selection, implementation, detection, followup, reporting, and other biases, with studies classified as high (5+ points), medium (3–4 points), or low quality (2 or fewer points) (Higgins et al., 2011).

### 2.5 Statistical analysis

Meta-analysis used Review Manager 5.3 and Stata 18.0. Standardized mean difference (SMD) and 95% confidence intervals (CI) were calculated. Significance was set at P < 0.05. Heterogeneity was assessed via Q-test (α = 0.1) (Hatala et al., 2005). A fixed-effects model was used if I2 ≤ 50%; otherwise, a random-effects model was applied, with subgroup, sensitivity, and meta-regression analyses to explore heterogeneity. Egger’s test assessed publication bias (Aviv, 2002).

## 3 Results

### 3.1 Literature search results

**FIGURE 2:**
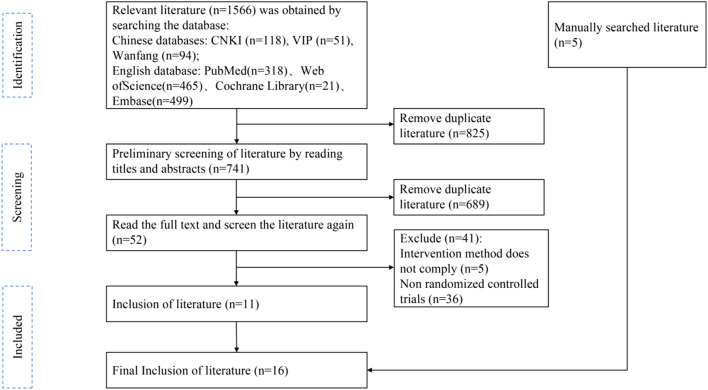
Flow Diagram of literature selection.

A total of 1,566 papers were initially obtained by searching various databases, including Chinese databases (CNKI, Wanfang, VIP) and English databases (PubMed, Web of Science, Cochrane Library, Embase). After importing them into EndNote X9 literature management software to remove duplicate papers, 741 papers remained. Preliminary screening by reading the titles and abstracts led to the exclusion of 689 irrelevant papers, leaving 52 papers. Following further full-text review, 41 papers were excluded due to intervention methods not complying (n = 5) or being non-randomized controlled trials (n = 36). Additionally, 5 manually searched literature pieces were added. Ultimately, 16 randomized controlled trial (RCT) papers were included in the qualitative and meta-analyses (Figure 2).

### 3.2 Basic characteristics and quality evaluation of the included papers

The basic characteristics of the 16 papers included in the Meta-analysis of this study are shown in Table 1. A total of 1,908 subjects were included in the Meta-analysis, with 1,005 in the experimental group and 903 in the control group. Among them, 11 papers adopted aerobic exercise (AE) intervention, 1 paper used high intensity interval training (HIIT) intervention, 3 papers applied resistance exercise (RE) intervention, and 3 paper used a combination of aerobic and resistance exercise intervention. The control groups in all included papers did not undergo any exercise intervention. The participants varied in type, including patients with breast cancer, women suffering from intimate partner violence, healthy women, people with high stress and lack of exercise, healthy populations, menopausal women, healthy elderly people, overweight and obese women, chronic disease patients, obese middle-aged females, postmenopausal women, PCOS women, myocardial infarction patients, and healthy older women. Gender distribution varied across studies, with some focusing on females, males, or mixed populations. Exercise intervention durations ranged from 8 to 52 weeks, with frequencies from 2 to 7 times per week.

**TABLE 1:** Characteristics of the studies included in the Meta⁃analysis.

Study	Country	Participants				Intervention					Outcome	Research quality/ score
		Type	Age/y	N	Gender (M/F)	Method	Intensity	Time / min·times^-1^	Frequency / Times/ week	Time/ weeks		
(Brown et al., 2023)	U.S.A	Patients with breast cancer	T 58.9 ± 8.4 C 59.2 ± 8.1	86 88	0/154	AE + RE	10RM/Moderate intensity	2–3 sets /30	2/3-6	52	①	4
(Cheung et al., 2019)	China	Women who suffer from intimate partner violence	T42.0±8.7 C 41.5 ± 9.3	136 135	0/271	AE	Baduanjin	30	7	22	②	6
(Eigendorf et al., 2019)	Germany	Healthy women	T 53.0±4.9 C 52.8±4.7	146 145	0/291	AE	—	20	3	24	①	4
(Puterman et al., 2018)	U.S.A	People with high stress and lack of exercise	T 59.3 ± 5.7 C63.3±6.4	34 34	13/55	AE	Low intensity - medium high intensity	20–30	3–5	16	①②	6
(Werner et al., 2019)	Germany	Healthy population	T 50.2±7.4 49.5±7.0 48.4±6.5 C 48.1±7.5	26 29 34 35	45/79	AE HIIT RE	60%HRR 4 × 4 20RM	45	3	26	①②	4
(Friedenreich et al., 2018)	Canada	Lack of exercise and healthy menopausal women	T 60.4 C 60.0	99 113	0/212	AE	70–80%HRR	45	5	48	①	6
(Duan et al., 2016)	China	Healthy elderly people	T 59.6±5.6 C 59.9±5.7	43 37	32/48	AE	Yang’s Tai Chi	60	5	24	②	6
(Dimauro et al., 2016)	Italy	Healthy elderly people	T 72± 1 C 72± 1	10 10	10/10	RE	10-12 times	3–4 sets	2	12	①	3
(Mason et al., 2014)	U.S.A	Overweight and obese women	T 58.1±5.0 C 57.4±4.4	106 79	0/185	AE	70–85%HRR	45	5	48	①	4
(Ho et al., 2012)	China	Chronic disease patients	T 42.1±7.3 C 42.5±5.5	33 31	13/51	AE	Five element balance skill	30	7	16	②	4
(Shin et al., 2008)	Korea	Obese middle-aged female	46.8±6.4	8 8	0/16	AE	60% VO_2_R	45	3	24	①	3
(Hagstrom and Denham, 2018)	Australia	Postmenopausal women	T 60.4 C 60.0	99 113	0/212	AE	70%–80% HRR	45	5	48	①	6
(Ribeiro et al., 2021)	Brazil	PCOS women	T 28.5±5.8 C 29.0±4.3	58 29	0/87	AE	50%–60% HRR	30–60	3	16	①	4
(Saks et al., 2016)	Iran	Myocardial infarction patients	T 57.3 ± 5.6 C 58.4 ± 5.4	10 10	20/0	AE + RE	8-15RM/50%–60% HRR	1-3sets/30	3	8	①②	4
(Sanchez-Gonzalez et al., 2021)	Spain	Healthy older women	T 71.2± 4.3 C 72.7±4.1	33 41	0/74	AE + RE	—	—	3	24	①	3
(Hoodenand-Moghadam et al., 2020)	Iran	Healthy elderly men	T 66.3± 3.4 C 66.1± 3	15 15	30/0	RE	60% 1RM	4 sets of the 6 exercise circuits	3	12	②	5

T: experimental group C: control group; AE: aerobic exercise; RE: resistance exercise; HIIT: High-intensity interval exercise; HR: Heart rate.

HRR: heart rate reserve; VO_2_R: The difference between maximum VO_2_ and resting VO_2_; ①: TL; ②: TA; PCOS: polycystic ovary syndrome.

Table 2 outlines the cell/tissue types used for analysis and the methods for measuring TL and TA. Leukocytes were commonly used for TL measurement via qPCR, while PBMCs were frequently used for TA measurement through methods like PCR ELISA PLUS or TRAP ELISA. DNA extraction methods also varied, including kits such as QIAamp DNA Mini kit, PAXgene^TM^ Blood DNA Tube, or Macherey-Nagel NucleoMag Blood 200 μL kit.

**TABLE 2:** Extraction methods of the Studies Included in the Meta⁃analysis.

Study	Cell/tissue type	TL	TA	DNA
(Brown et al., 2023)	PBMCs, Lymphocyte	qPCR	—	PAXgene^TM^ Blood DNA Tube, BD Sciences
(Cheung et al., 2019)	PBMCs	—	PCR ELISAPLUS	ELISA
(Eigendorf et al., 2019)	PBMCs	qPCR	—	QIAamp DNA Mini kit
(Puterman et al., 2018)	PBMCs, Leukocytes	qPCR	ddPCR	QIAamp® DNA Blood Midi kit
(Werner et al., 2019)	PBMCs, Leukocytes	Flow cytometry, FISH, PCR	Lightcycler	QIAamp DNA Blood Mini Kit(Column extraction)
(Friedenreich et al., 2018)	PBMCs, Leukocytes	qPCR	—	Macherey-Nagel NucleoMag Blood 200 μL kit
(Duan et al., 2016)	PBMCs	—	TE ELISA	Sodium citrate tube
(Dimauro et al., 2016)	PBMCs	RT-PCR	—	ChargeSwitch gDNA 50–100 μL blood Kit
(Mason et al., 2014)	PBMCs, Leukocytes	qPCR	—	Qiagen Midi Kit Kit(Column extraction)
(Ho et al., 2012)	PBMCs	—	TRAP ELISA	Ficoll-Paque PLUS
(Shin et al., 2008)	PBMCs	qPCR	—	Wizard Genomic DNA Purification Kit
(Hagstrom and Denham, 2018)	PBMCs, Leukocytes	qPCR	—	Macherey-Nagel NucleoMag Blood 200 μL kit
(Ribeiro et al., 2021)	PBMCs, Leukocytes	qPCR	—	MasterPure Complete DNA and RNA Purification Kit
(Saks et al., 2016)	PBMCs	qPCR	qPCR	—
(Sanchez-Gonzalez et al., 2021)	Saliva	qPCR	—	NanoDropTM 2000/2001 spectrophotometer
(Hoodenand-Moghadam et al., 2020)	PBMCs	—	ELISA human kit	ELISA human kit

PBMCs:Peripheral blood mononuclear cells; qPCR: Quantitative Polymerase Chain Reaction; TRAP: Telomeric Repeat Amplification Protocol; ddPCR: Droplet Digital PCR; TE-ELISA: human telomerase–enzyme linked immunosorbent assay.

**FIGURE 3:**
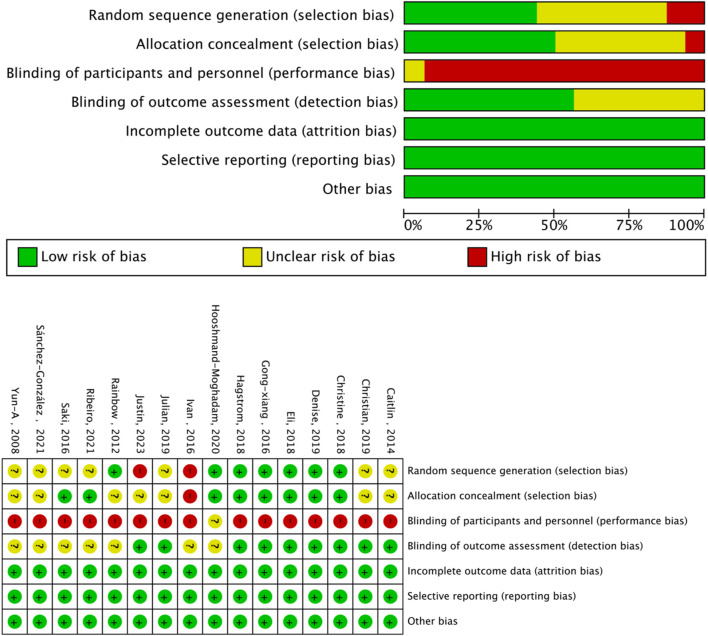
Analysis of the risk of bias in Accordance with the Cochrane Collaboration Guidelines.

**FIGURE 4:**
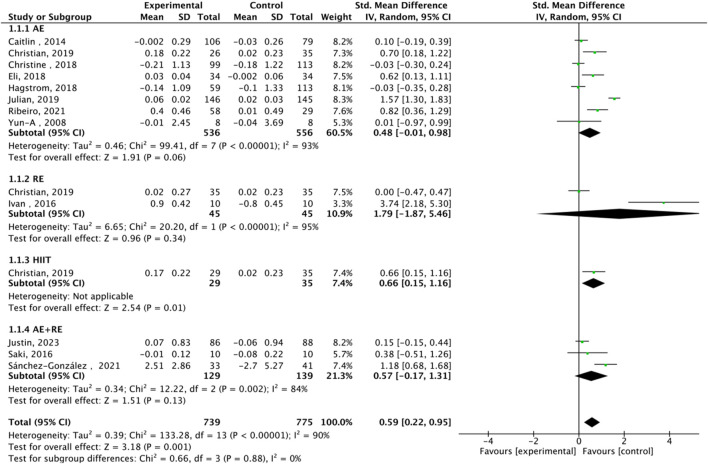
Subgroup analysis of TL effect size under Different Modes of exercise.

The Cochrane risk of bias assessment tool was used to evaluate the quality of the above papers. Six papers were of high quality, and nine were of medium quality. The evaluation results are shown in Figures 3, 4.

### 3.3 Meta-analysis results

#### 3.3.1 Meta-analysis of the effect size of TL

**FIGURE 5:**
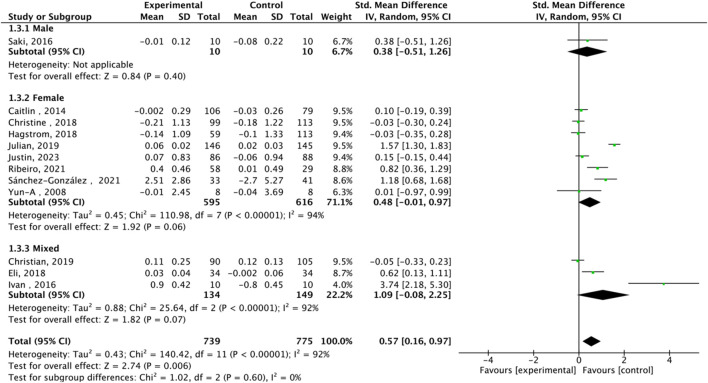
Subgroup analysis of TL effect size under Different gender of exercise.

**FIGURE 6 F6:**
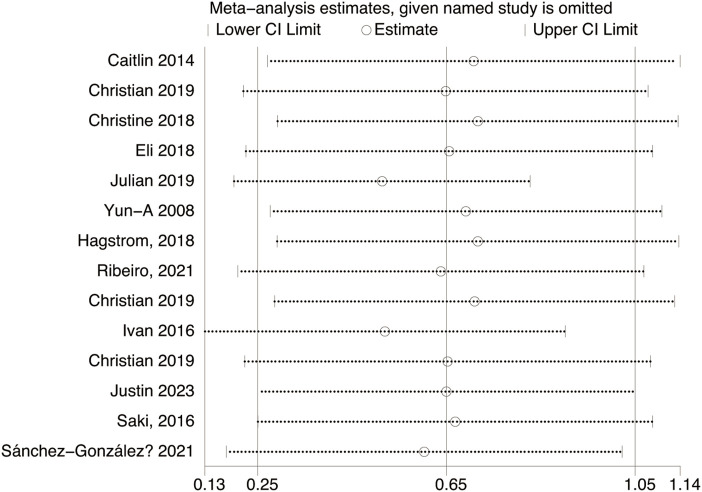
Sensitivity analysis of TL effect size under Different Modes of exercise.

Fourteen studies assessed TL. Exercise maintained TL (SMD = 0.59, 95% CI: 0.22–0.95, P = 0.001, I2 = 92%, random-effects model) (Figure 4). Subgroup analysis by exercise type showed trends for AE (SMD = 0.48, P = 0.06, I2 = 93%), RE (SMD = 1.79, P = 0.34, I2 = 95%), HIIT (SMD = 0.66, P = 0.01, single study), and AE + RE (SMD = 0.57, P = 0.13). The HIIT result is preliminary due to reliance on a single study. Subgroup analysis by sex showed a trend for females (SMD = 0.48, P = 0.06) over males (SMD = 0.38, P = 0.40) (Figure 5). Sensitivity analysis indicated stable results (Figure 6). Meta-regression identified publication year (2016–2018) as a heterogeneity source (β = −1.256, P = 0.026) (Table 3)

**TABLE 3:** Meta-regression analysis results of heterogeneity factors Affecting TL effect size.

Research features	Regression coefficient(β)	95%CI	t	p
Intervention time	−0.04	−0.08∼0.008	−1.91	0.09
Sample size	−0.002	−0.01∼0.01	−0.39	0.71
health	0.69	−0.79∼2.16	1.07	0.32
country	0.40	−0.21∼1.00	1.51	0.17
Gender	0.56	−0.95∼2.07	0.85	0.42
Article quality	−0.45	−2.34∼1.44	−0.55	0.60
Publication Year 2016–2018	−1.25628	−2.32∼−0.20	−2.73	0.026

#### 3.3.2 Meta - analysis of the effect size of TA

**FIGURE 7:**
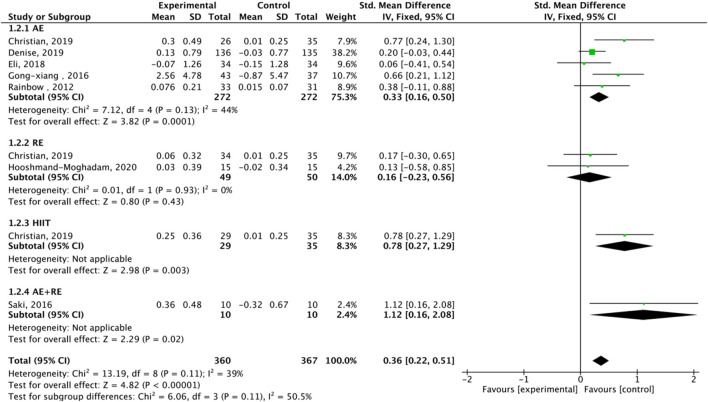
Subgroup analysis of TA effect size under different modes of exercise.

**FIGURE 8:**
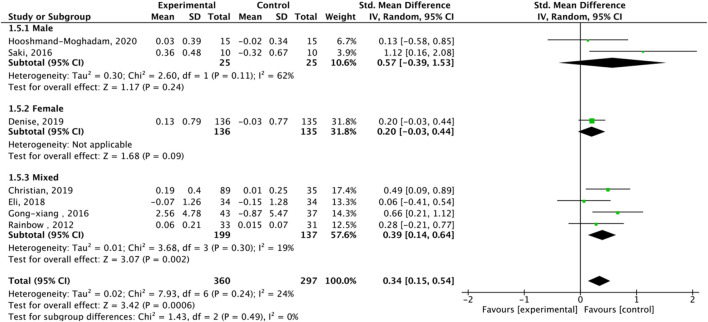
Subgroup analysis of TA effect size under different gender of exercise.

Nine studies assessed TA. Exercise enhanced TA (SMD = 0.36, 95% CI: 0.22–0.51, P < 0.00001, I2 = 39%, fixed-effects model) (Figure 7). Subgroup analysis showed significant effects for AE (SMD = 0.33, P = 0.0001, I2 = 44%) and HIIT (SMD = 0.78, P = 0.003, single study), but not RE (SMD = 0.16, P = 0.43). Mixed-gender groups showed significant TA increases (SMD = 1.12, P = 0.02) (Figure 8).

#### 3.3.3 Publication bias analysis

**FIGURE 9:**
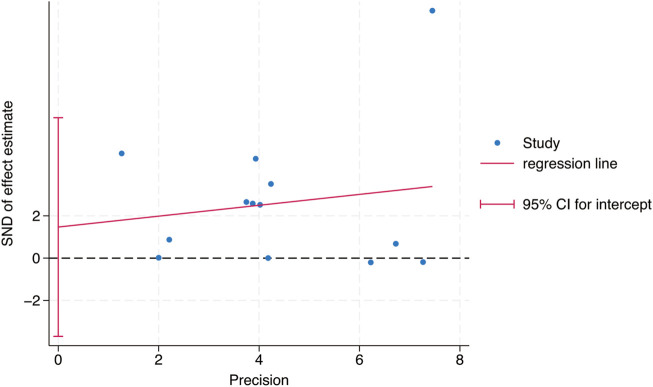
Bias analysis of the impact of exercise intervention on TL

**FIGURE 10:**
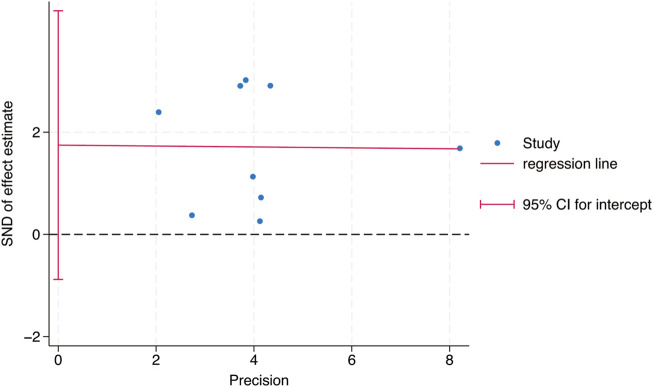
Bias analysis of the impact of exercise intervention on TA

Egger’s test was used to study the publication bias of the literature from two aspects: the intervention effect of exercise on TL and TA. When the intercept segment crossed the zero point, the publication bias was low. For the intervention effect of exercise on TL, the test result was t = 0.46, P = 0.66, 95% CI: (−5.42–8.15), which included 0, indicating that there was no obvious publication bias in the intervention effect of exercise on TL, and the results of the Meta - analysis were relatively stable. For the intervention effect of exercise on TA, the test result was t = 1.35, P = 0.24, 95% CI: (−1.91–6.11), which included 0, indicating that there was no obvious publication bias in the intervention effect of exercise on TA, and the results of the Meta - analysis were relatively stable (Figures 9, 10).

## 4 Discussion

Exercise maintains TL and enhances TA, potentially contributing to delayed aging. This meta-analysis of 16 RCTs provides evidence for exercise prescriptions targeting telomere protection, aligning with prior meta-analyses like Schellnegger et al. (2022), which found exercise associated with longer TL in leukocytes (SMD = 0.41, P < 0.05) but noted similar heterogeneity challenges (Schellnegger et al., 2022). TL and TA are robust biomarkers of cellular aging, reflecting replication potential more directly than oxidative stress or inflammatory markers (Tacutu et al., 2013). Exercise maintained TL (SMD = 0.60, P = 0.01) and enhanced TA (SMD = 0.35, P < 0.00001). The claim of telomere lengthening is tempered by mechanisms such as selective apoptosis of cells with short telomeres, which may increase the proportion of cells with longer telomeres without actual elongation (Beyne-Rauzy et al., 2005). Thus, exercise primarily maintains TL relative to sedentary controls. TA increases may result from telomerase recruitment to short telomeres (Zou et al., 2004), immune cell proliferation (Simpson et al., 2010), or upregulation of TERT expression (Zhang J. et al., 2016). Mechanistically, exercise reduces oxidative stress via enhanced antioxidant enzyme activity (e.g., superoxide dismutase) (Shin et al., 2008) and suppresses inflammation through reduced pro-inflammatory cytokines (e.g., IL-6, TNF-α) (Werner et al., 2019; von Zglinicki, 2002), both of which protect telomeres from damage (von Zglinicki, 2002).

Subgroup analysis by sex showed a stronger TL maintenance trend in females (SMD = 0.48, P = 0.06) than males (SMD = 0.38, P = 0.40), possibly due to estrogen’s role in telomerase regulation (Konar et al., 2016). AE consistently enhanced TA (SMD = 0.33, P = 0.0001), while HIIT showed promise for TL maintenance (SMD = 0.66, P = 0.01), though this finding is limited by a single study (Werner et al., 2019). RE showed non-significant trends (SMD = 0.16, P = 0.43), likely due to only three studies and high variability in protocols (e.g., intensity, volume) (Zhang F. et al., 2016). Merging AE and RE categories was considered but not implemented, as their distinct physiological mechanisms (e.g., oxidative stress reduction in AE vs muscle hypertrophy in RE) justify separate analyses (Vakonaki et al., 2018).

High heterogeneity (I2 = 92% for TL) was partially explained by measurement methods (e.g., qPCR, Flow-FISH, Southern blot), participant age, and baseline health (Table 2). For example, qPCR is less precise than Southern blot for TL measurement, potentially inflating variability (Chakrabarti and Mohanakumar, 2016). TRAP ELISA for TA is less reliable than gel-based TRAP or droplet digital PCR (Friedenreich et al., 2018). Participant diversity (healthy, cancer, obese, stressed) and age (20–80 years) likely amplify heterogeneity, as disease states or older age may enhance exercise effects (Puterman et al., 2018). Metaregression identified publication year as a significant heterogeneity source, but only 25.2% of variance was explained, suggesting unexamined confounders like diet or genetics (von Zglinicki, 2002). The forest plots (Figures 4,6, [Fig F6]) correctly represent effect sizes favoring exercise, with positive SMD indicating TL/TA increases.

Causal claims about exercise delaying aging are tempered by potential confounders. Diet (e.g., antioxidant intake) and genetic factors (e.g., TERT polymorphisms) may influence TL and TA independently or interact with exercise effects (von Zglinicki, 2002). For instance, high antioxidant diets may synergize with exercise to reduce oxidative stress, while genetic predispositions may modulate telomerase response (de Lange, 2005). These factors were not controlled in most included studies, limiting causal inferences.

Exercise prescriptions include:

• TL maintenance: HIIT, ≥16 weeks, ≥60 min/week, 80%–90% max heart rate, pending further validation.
• TA enhancement: AE (e.g., running, swimming), ≥150 min/week, 60%–75% heart rate reserve, ≥6 months.
• Comprehensive strategy: Combine AE and RE (e.g., Taijiquan) for synergistic effects (Blackburn, 2001).

Limitations include reliance on English literature, limited HIIT/RE studies, measurement variability, and uncontrolled confounders like diet and genetics. Compared to Schellnegger et al. (2022), our study includes more recent RCTs and TA outcomes but faces similar heterogeneity challenges (Schellnegger et al., 2022). Future research should standardize TL/TA measurement methods (e.g., adopt Southern blot or droplet digital PCR), control for confounders, and explore sex- and cell-specific effects.

## 5 Conclusion

Exercise maintains TL and enhances TA, potentially contributing to delayed aging. AE shows robust effects on TA, while HIIT and RE require further research due to limited studies and non-significant results for RE. Standardized measurement methods and control for confounders like diet and genetics are needed to strengthen causal inferences.

## Data Availability

The original contributions presented in the study are included in the article/supplementary material, further inquiries can be directed to the corresponding authors.

## Glossary

RCTs: randomized controlled trials
TRF1: telomeric repeat binding factor 1
TRF2: telomeric repeat binding factor 2
POT1: protection of telomeres 1 protein
TIN2: TRF1 - and TRF2 - interacting nuclear protein 2
TPP1: TIN2 and POT1 interacting protein 1
RAP1: repressor activator protein 1
TERT: telomerase reverse transcriptase
TERC: telomerase RNA template
AE: Aerobic exercise
RE: Resistance exercise
HIIT: High-intensity interval exercise
HR: Heart rate

## References

[B1] Arnoult N. Karlseder J. (2015). Complex interactions between the DNA-Damage response and Mammalian telomeres. Nat. Struct. Mol. Biol. 22 (11), 859–866. 10.1038/nsmb.3092 26581520 PMC4739752

[B2] Aviv A. (2002). Telomeres, sex reactive oxygen species, and human cardiovascular aging. J. Mol. Med. Berl. 80 (11), 689–695. 10.1007/s00109-002-0377-8 12436345

[B3] Balan E. Decottignies A. Deldicque L. (2018). Physical activity and nutrition: two promising strategies for telomere maintenance? Nutrients 10 (12), 1942. 10.3390/nu10121942 30544511 PMC6316700

[B4] Beyne-Rauzy O. Prade-Houdellier N. Demur C. Recher C. Ayel J. Laurent G. (2005). Tumor necrosis factor-alpha inhibits hTERT gene expression in human myeloid normal and leukemic cells. Blood 106 (9), 3200–3205. 10.1182/blood-2005-04-1386 16020509

[B5] Blackburn E. H. (2001). Switching and signaling at the telomere. Cell 106 (6), 661–673. 10.1016/s0092-8674(01)00492-5 11572773

[B6] Blackburn E. H. (2010). Telomeres and telomerase: the means to the end (nobel lecture). Angew. Chem. Int. Ed. Engl. 49 (41), 7405–7421. 10.1002/anie.201002387 20821774

[B7] Blackburn E. H. Epel E. S. Lin J. (2015). Human telomere biology: a contributory and interactive factor in aging, disease risks, and protection. Science 350 (6265), 1193–1198. 10.1126/science.aab3389 26785477

[B8] Brown J. C. Sturgeon K. Sarwer D. B. Troxel A. B. DeMichele A. M. Denlinger C. S. (2023). The effects of exercise and diet on oxidative stress and telomere length in breast cancer survivors. Breast Cancer Res. Treat. 199 (1), 109–117. 10.1007/s10549-023-06868-5 36933050 PMC10148914

[B9] Brown L. Needham B. Ailshire J. (2017). Telomere length among older U.S. adults: differences by race/ethnicity, gender, and age. J. Aging Health 29 (8), 1350–1366. 10.1177/0898264316661390 27469599 PMC5272874

[B10] Chakrabarti S. Mohanakumar K. P. (2016). Aging and neurodegeneration: a tangle of models and mechanisms. Aging Dis. 7 (2), 111–113. 10.14336/AD.2016.0312 27114843 PMC4809602

[B11] Cheung D. S. T. Deng W. Tsao S. W. Ho R. T. H. Chan C. L. W. Fong D. Y. T. (2019). Effect of a qigong intervention on telomerase activity and mental health in Chinese women survivors of intimate partner violence: a randomized clinical trial. JAMA Netw. Open 2 (1), e186967. 10.1001/jamanetworkopen.2018.6967 30646209 PMC6484539

[B12] Cleal K. Norris K. Baird D. (2018). Telomere length dynamics and the evolution of cancer genome architecture. Int. J. Mol. Sci. 19 (2), 482. 10.3390/ijms19020482 29415479 PMC5855704

[B13] Cong Y. S. Wright W. E. Shay J. W. (2002). Human telomerase and its regulation. Microbiol. Mol. Biol. Rev. 66 (3), 407–425. table of contents. 10.1128/MMBR.66.3.407-425.2002 12208997 PMC120798

[B14] de Lange T. (2005). Shelterin: the protein complex that shapes and safeguards human telomeres. Genes Dev. 19 (18), 2100–2110. 10.1101/gad.1346005 16166375

[B15] Dimauro I. Scalabrin M. Fantini C. Grazioli E. Beltran Valls M. R. Mercatelli N. (2016). Resistance training and redox homeostasis: correlation with age-associated genomic changes. Redox Biol. 10, 34–44. 10.1016/j.redox.2016.09.008 27687219 PMC5040637

[B16] Duan G.-x. Wang K. Su Y.-h. Tang S.-y. Jia H.-l. Chen X.-m. (2016). Effects of Tai chi on telomerase activity and gerotranscendence in middle aged and elderly adults in Chinese society. Int. J. Nurs. Sci. 3, 235–241. 10.1016/j.ijnss.2016.07.005

[B17] Eigendorf J. Melk A. Haufe S. Boethig D. Berliner D. Kerling A. (2019). Effects of personalized endurance training on cellular age and vascular function in middle-aged sedentary women. Eur. J. Prev. Cardiol. 26 (17), 1903–1906. 10.1177/2047487319849505 31084260

[B18] Friedenreich C. M. Wang Q. Ting N. S. Brenner D. R. Conroy S. M. McIntyre J. B. (2018). Effect of a 12-month exercise intervention on leukocyte telomere length: results from the ALPHA trial. Cancer Epidemiol. 56, 67–74. 10.1016/j.canep.2018.07.012 30075329

[B19] Hagstrom A. D. Denham J. (2018). The effect of resistance training on telomere length in women recovering from breast cancer. J. Strength Cond. Res. 32, 1589–1596. 10.3390/jfmk3010009

[B20] Harley C. B. Futcher A. B. Greider C. W. (1990). Telomeres shorten during ageing of human fibroblasts. Nature 345 (6274), 458–460. 10.1038/345458a0 2342578

[B21] Hatala R. Keitz S. Wyer P. Guyatt G. Evidence-Based Medicine Teaching Tips Working Group (2005). Tips for learners of evidence-based medicine: 4. Assessing heterogeneity of primary studies in systematic reviews and whether to combine their results. Cmaj 172 (5), 661–665. 10.1503/cmaj.1031920 15738493 PMC550638

[B22] Helbig S. Wockner L. Bouendeu A. Hille-Betz U. McCue K. French J. D. (2017). Functional dissection of breast cancer risk-associated TERT promoter variants. Oncotarget 8 (40), 67203–67217. 10.18632/oncotarget.18226 28978027 PMC5620167

[B23] Hemann M. T. Strong M. A. Hao L. Y. Greider C. W. (2001). The shortest telomere, not average telomere length, is critical for cell viability and chromosome stability. Cell 107 (1), 67–77. 10.1016/s0092-8674(01)00504-9 11595186

[B24] Higgins J. P. Altman D. G. Gøtzsche P. C. Jüni P. Moher D. Oxman A. D. (2011). The cochrane Collaboration's tool for assessing risk of bias in randomised trials. Bmj 343, d5928. 10.1136/bmj.d5928 22008217 PMC3196245

[B25] Ho R. T. Chan J. S. Wang C. W. Lau B. W. So K. F. Yuen L. P. (2012). A randomized controlled trial of qigong exercise on fatigue symptoms, functioning, and telomerase activity in persons with chronic fatigue or chronic fatigue syndrome. Ann. Behav. Med. 44 (2), 160–170. 10.1007/s12160-012-9381-6 22736201 PMC3442161

[B26] Hoodenand-Moghadam M. Najafi A. Mohammadi M. Malekzadeh J. M. (2020). Effect of aerobic training on telomerase activity in peripheral blood mononuclear cells of middleaged men. J. Biol. Regul. Homeost. Agents 34, 1895–1901. 10.23812/20-123-L

[B27] Kenyon C. J. (2010). The genetics of ageing. Nature 464 (7288), 504–512. 10.1038/nature08980 20336132

[B28] Konar A. Singh P. Thakur M. K. (2016). Age-associated cognitive decline: insights into molecular switches and recovery avenues. Aging Dis. 7 (2), 121–129. 10.14336/AD.2015.1004 27114845 PMC4809604

[B29] López-Otín C. Blasco M. A. Partridge L. Serrano M. Kroemer G. (2013). The hallmarks of aging. Cell 153 (6), 1194–1217. 10.1016/j.cell.2013.05.039 23746838 PMC3836174

[B30] Mason C. Risques R. A. Xiao L. Duggan C. R. Imayama I. Campbell K. L. (2014). Independent and combined effects of dietary weight loss and exercise on leukocyte telomere length in postmenopausal women. Obes. (Silver Spring) 21 (12), E549–E554. 10.1002/oby.20509 PMC3786031 23640743

[B31] Mercken E. M. Carboneau B. A. Krzysik-Walker S. M. de Cabo R. (2012). Of mice and men: the benefits of caloric restriction, exercise, and mimetics. Ageing Res. Rev. 11 (3), 390–398. 10.1016/j.arr.2011.11.005 22210414 PMC3356510

[B32] Puterman E. Weiss J. Lin J. Schilf S. Slusher A. L. Johansen K. L. (2018). Aerobic exercise lengthens telomeres and reduces stress in family caregivers: a randomized controlled trial - curt richter award paper 2018. Psychoneuroendocrinology 98, 245–252. 10.1016/j.psyneuen.2018.08.002 30266522

[B33] Ribeiro J. Oliveira A. Silva I. Santos C. Duarte J. A. (2021). Effects of exercise on leukocyte telomere length in healthy adults: a randomized controlled trial. Eur. J. Sport Sci. 21, 567–575. 10.1080/17461391.2020.1754467

[B34] Saks E. Tiit M. Veldre G. Riso E. M. Märtson A. Jürimäe J. (2016). Exercise and telomere length in peripheral blood mononuclear cells in older adults. Clin. Sci. 130, 1357–1366. 10.1042/CS20160058

[B35] Sanchez-Gonzalez J. L. Sanchez-Rodriguez J. L. Martin-Vallejo J. Martel-Martel A. Gonzalez-Sarmiento R. (2021). Effects of an exercise program on salivary telomere length and stress biomarkers in schoolchildren. Oral Dis. 27, 629–636. 10.1111/odi.13594

[B36] Schellnegger M. Lin A. C. Hammer N. Kamolz L. P. (2022). Physical activity on telomere length as a biomarker for aging: a systematic review. Sports Med. Open 8, 111. 10.1186/s40798-022-00503-1 36057868 PMC9441412

[B37] Shin Y. A. Lee J. H. Song W. Jun T. W. (2008). Exercise training improves the antioxidant enzyme activity with no changes of telomere length. Mech. Ageing Dev. 129 (5), 254–260. 10.1016/j.mad.2008.01.001 18295822

[B38] Simpson R. J. Cosgrove C. Ingram L. A. Florida-James G. D. Whyte G. P. Pircher H. (2010). Senescent T-lymphocytes are mobilised into the peripheral blood compartment in young and older humans after exhaustive exercise. Exerc Immunol. Rev. 16, 96–116. 10.1016/j.bbi.2007.11.002 18164586

[B39] Stambler I. (2017). Recognizing degenerative aging as a treatable medical condition: methodology and policy. Aging Dis. 8 (5), 583–589. 10.14336/AD.2017.0130 28966803 PMC5614323

[B40] Tacutu R. Craig T. Budovsky A. Wuttke D. Lehmann G. Taranukha D. (2013). Human ageing genomic resources: integrated databases and tools for the biology and genetics of ageing. Nucleic Acids Res. 41 (Database issue), D1027–D1033. 10.1093/nar/gks1155 23193293 PMC3531213

[B41] Vakonaki E. Tsiminikaki K. Plaitis S. Fragkiadaki P. Tsoukalas D. Katsikantami I. (2018). Common mental disorders and association with telomere length. Biomed. Rep. 8 (2), 111–116. 10.3892/br.2018.1040 29435268 PMC5778888

[B42] von Zglinicki T. (2002). Oxidative stress shortens telomeres. Trends Biochem. Sci. 27, 339–344. 10.1016/S0968-0004(02)02110-2 12114022

[B43] Werner C. M. Hecksteden A. Morsch A. Zundler J. Wegmann M. Kratzsch J. (2019). Differential effects of endurance, interval, and resistance training on telomerase activity and telomere length in a randomized, controlled study. Eur. Heart J. 40 (1), 34–46. 10.1093/eurheartj/ehy585 30496493 PMC6312574

[B44] Zhang F. Cheng D. Wang S. Zhu J. (2016a). Human specific regulation of the telomerase reverse transcriptase gene. Genes (Basel) 7 (7), 30. 10.3390/genes7070030 27367732 PMC4962000

[B45] Zhang J. Rane G. Dai X. Shanmugam M. K. Arfuso F. Samy R. P. (2016b). Ageing and the telomere connection: an intimate relationship with inflammation. Ageing Res. Rev. 25, 55–69. 10.1016/j.arr.2015.11.006 26616852

[B46] Zou Y. Sfeir A. Gryaznov S. M. Shay J. W. Wright W. E. (2004). Does a sentinel or a subset of short telomeres determine replicative senescence? Genes Dev. 18, 3074–3085. 10.1101/gad.1252104 PMC491830 15181152

